# Toward Gamified Pain Management Apps: Mobile Application Rating Scale–Based Quality Assessment of Pain-Mentor’s First Prototype Through an Expert Study

**DOI:** 10.2196/13170

**Published:** 2020-05-26

**Authors:** Alexandra Hoffmann, Corinna A Faust-Christmann, Gregor Zolynski, Gabriele Bleser

**Affiliations:** 1 Junior Research Group wearHEALTH Department of Computer Science Technische Universität Kaiserslautern Kaiserslautern Germany

**Keywords:** mHealth, chronic pain, stress management, pain management, health app, gamification, health professional

## Abstract

**Background:**

The use of health apps to support the treatment of chronic pain is gaining importance. Most available pain management apps are still lacking in content quality and quantity as their developers neither involve health experts to ensure target group suitability nor use gamification to engage and motivate the user. To close this gap, we aimed to develop a gamified pain management app, Pain-Mentor.

**Objective:**

To determine whether medical professionals would approve of Pain-Mentor’s concept and content, this study aimed to evaluate the quality of the app’s first prototype with experts from the field of chronic pain management and to discover necessary improvements.

**Methods:**

A total of 11 health professionals with a background in chronic pain treatment and 2 mobile health experts participated in this study. Each expert first received a detailed presentation of the app. Afterward, they tested Pain-Mentor and then rated its quality using the mobile application rating scale (MARS) in a semistructured interview.

**Results:**

The experts found the app to be of excellent general (mean 4.54, SD 0.55) and subjective quality (mean 4.57, SD 0.43). The app-specific section was rated as good (mean 4.38, SD 0.75). Overall, the experts approved of the app’s content, namely, pain and stress management techniques, behavior change techniques, and gamification. They believed that the use of gamification in Pain-Mentor positively influences the patients’ motivation and engagement and thus has the potential to promote the learning of pain management techniques. Moreover, applying the MARS in a semistructured interview provided in-depth insight into the ratings and concrete suggestions for improvement.

**Conclusions:**

The experts rated Pain-Mentor to be of excellent quality. It can be concluded that experts perceived the use of gamification in this pain management app in a positive manner. This showed that combining pain management with gamification did not negatively affect the app’s integrity. This study was therefore a promising first step in the development of Pain-Mentor.

## Introduction

### Background

Approximately one-third of the American and European population suffers from chronic pain [1,2]. This makes chronic pain a major health care problem that needs to be taken more seriously [2]. With profound negative consequences on psychological, social, physical, and economic aspects for those affected, chronic pain can have a serious negative impact on a person’s overall quality of life [3-6].

Next to medical treatments (eg, medication, surgical rehabilitation, and physical therapy), psychological treatments are an important aspect of pain management [1].

In fact, the combination of 5 theory-based functionalities, namely, pain-related education, self-monitoring, goal setting, social support, and the training of self-care strategies, has been suggested to promote the self-management of chronic pain [7-11]. As patients need to understand and manage the thoughts, emotions, and behaviors that often accompany chronic pain [1], the mediated self-care strategies should include stress-coping skills such as relaxation techniques, problem solving, and communication skills training [12-14]. Multimodal approaches that integrate these aspects can improve the overall quality of life in patients with chronic pain, as compared with treatments that are strictly medication focused [15-17].

However, the integration of multimodal approaches into routine primary and tertiary care has been slow [2]. Major barriers such as poor accessibility because of geographical reasons, limited availability of trained professionals, and high therapy-related costs keep patients from accessing pain-specific education and psychological treatment [18-20]. As a result, most patients never receive the required education or skills training to promote self-management of pain [19-21].

However, the care for chronic pain is no longer strictly limited to medical environments and clinician-guided telehealth because of the rising number of mobile health (mHealth) products [22]. mHealth describes the use of mobile technology to improve health [23] by affecting the user’s education, motivation, and adherence [24,25]. It has already been applied to support mental as well as physical health programs [26]. As such, mHealth can enhance the self-management of chronic conditions [27]. Indeed, preliminary evidence suggests that pain management apps have great potential to support chronic pain treatment and are well received by patients [12,28] and health care professionals [29]. In fact, a majority of studies reviewed by Thurnheer et al [29] showed beneficial effects of the use of pain management apps on pain severity. As such apps are available anywhere, anytime [30], they can reduce the frequency and cost of face-to-face interventions [31]. Moreover, they can combine a variety of features (eg, educational content, a diary, personalized recommendations, and communication with health care professionals) within one app [29]. As a result, they have the potential to make health care systems more effective [31].

To ensure their effectiveness, apps for chronic pain management must be based on evidence-based content (ie, pain-related education, self-monitoring, goal setting, social support, and the training of self-care strategies including stress management) [23,32,33]. Even though these aspects are easy enough to implement, app reviews show that existing pain management apps have limited content. Rather than providing evidence-based behavior change programs, the reviewed pain management apps reveal a lack of combination of evidence-based functionalities [34-36]. Indeed, most apps only include 1 of the 5 suggested components [34,36]. Apps mostly focus on supplying information [34-36]. They seldom help to achieve social support and often lack evidence-based self-care skills and tracking of the multidimensional experience of pain. Most apps only allow to track pain intensity (eg, FitBack [12]) [34,35,37,38]. Some apps also allow the user to track other pain-related aspects such as pain location, medication, and pain source (eg, Pain Squat [18]). However, only a small number of apps also allow the assessment of emotional and cognitive aspects. In addition, the educational content that is included is often of poor quality. An exception is the pain diary app, PainTracker, which includes 3 of the 5 suggested functionalities. It allows the tracking of a number of pain-related aspects, allows goal setting, and provides informational content [28]. Notwithstanding this exception, the overall lack of content in available pain management apps leads to a distrust in their effectiveness [39]. As a result, comprehensive, evidence-based, and clinically informed smartphone apps for pain self-management are highly needed [35].

Although important, the use of evidence-based content alone has been considered as insufficient to ensure adequate user engagement and motivation [40], two aspects that have great influence on the usage of an intervention program [41]. In fact, further improvement is needed to make pain management apps more engaging and entertaining [28].

One way to increase user engagement and motivation is through gamification. Gamification, the use of game elements in nongame contexts [42], aims to make interventions, such as health apps, more enjoyable, motivating, and engaging [43]. However, the use of gamification in health apps has been critically discussed as its effects depend on the context and the goal of the app [44]. Although users do not always want the implementation of gamification in health apps [25], it could be shown that its use can have positive effects on both health and wellness [44].

Gamification has already been shown to positively influence user self-management [43], lifestyle [45], health and behavioral outcomes [46], and the retention of desired user behaviors [47]. This confirms that the implementation of gamification can be effective in promoting behavior change through apps [48].

Nevertheless, few pain management apps make use of this concept. Two pain management apps for adolescent patients with cancer have included gamification in the form of a virtual rewards system and ranks that are linked to the users’ pain diaries and adherence [18,49]. However, to our knowledge, there is no chronic pain management app with an extensive gamification framework for adults.

### Pain-Mentor

As current pain management apps lack in their use of both evidence-based self-management skills [35] and gamification [28], we aimed to develop Pain-Mentor to close this gap in research. Pain management apps have great potential to promote patient self-care in out-clinic settings [29]. Therefore, Pain-Mentor supports the therapy of patients with chronic pain by teaching evidence-based techniques from multi-modal pain therapy and how they can be applied in everyday life. The app is based on the concept of Stress-Mentor [50], a gamified stress management app. Stress-Mentor includes all 5 suggested theory-based functionalities. For example, it realizes self-monitoring through a diary that allows the tracking of up to 14 diary categories (ie, sleep duration and quality, sport duration and intensity, daily uplifts and daily hassles, stress level, mood, digestion, consumption of water, fruits and vegetables, coffee and alcohol, and step count). In addition, the app teaches different self-help skills through daily and weekly tasks. In these tasks, the user can choose 1 of 3 suggested skills that he or she wants to practice. The techniques offered by the app depend on the user’s entries into a stress checklist. In the stress checklist, the user can enter stress-related aspects (ie, fears and worries, sadness, anger, stress at work, stress in private life, muscle tension, and head, neck, and back grievances caused by tension, digestive problems, and sleep problems) on a scale of 0 to 10 on a weekly basis. This concept of personalized tasks encourages the user to set daily and weekly goals and supports the repetition of exercises. The mediated skills include relaxation exercises (ie, abdominal breathing, meditation, mindfulness, progressive muscle relaxation, guided imagery, stretching exercises, and self-massage), problem solving (ie, time management, goal setting, planning of social support and social change, and barrier identification), cognitive aspects (ie, assertiveness training, refuting irrational ideas, appraisal of stress and stressful situations, and avoiding perfectionism), and the transfer of educational information about stress. Moreover, the app provides tips on stress based on the user’s documented stress level.

In addition to self-monitoring, stress management skills, and educational information, Stress-Mentor includes several other behavior change techniques (see the study by Christmann et al [50] for a detailed list) to support long-term behavior change. The included behavior change techniques are linked to an extensive gamification concept aimed at motivating and engaging the user [50]. As such, the app includes an avatar (a bird-like cartoon animal) that provides feedback by reflecting both the user’s diary entries (vicarious reinforcement) [51,52] and progress. Another aspect is the app’s agent (a wise owl), who is a mentor that entrusts the care of the avatar to the user via a behavioral contract and provides instructions on app functions, general encouragement, and educational tips on stress. The user can collect *woodland coins* that can later be exchanged for items for the user’s avatar. Moreover, the app provides feedback on the user’s performance through progress bars, a diary overview diagram [53], badges, and the visual development of the avatar and its surroundings. A detailed description of the implemented behavior change techniques and how they were linked with gamification was previously published [50].

**Figure 1:**
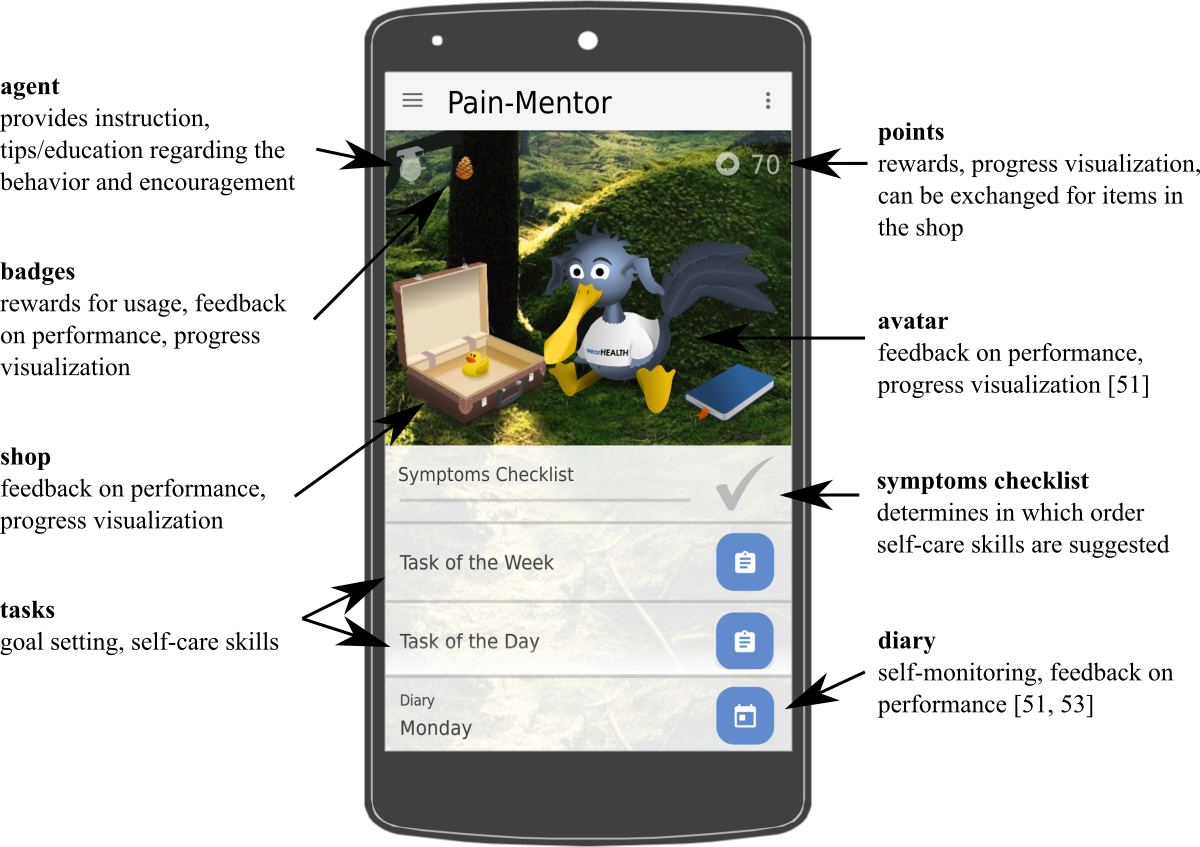
Screenshot of Pain-Mentor.

As stress management and cognitive and behavioral aspects play an important role in the treatment of chronic pain [1], Stress-Mentor’s concept was adopted for the first prototype of the pain management app (Pain-Mentor) evaluated in this study. Although all stress-related content remains, additions were made to the existing diary, tips, symptoms checklist, and daily tasks to further adapt Pain-Mentor to the context of chronic pain treatment. We made the following adjustments to better suit Pain-Mentor to its designated usage context. First, the stress checklist was renamed to symptoms checklist. The symptoms checklist was then extended with a numerical rating scale for pain that is commonly used in therapy. It allows the user to enter his or her pain level on a scale of 0 (no pain) to 10 (worst possible pain) [54]. The diary was also extended by this scale. This provides users with the opportunity to track the trend of their pain on a daily basis [55]. A total of 8 pain-specific daily tasks were added to the task pool: 1 each to develop a plan for setbacks, planning social support for pain management, and planning a dropped activity and 5 physical exercises for muscle strengthening and stretching. Moreover, the tips given by the app’s mentor were extended with additional information on chronic pain and pain management. A screenshot of the app is displayed in Figure 1.

All in all, Pain-Mentor differs from other pain management apps regarding one important property: it includes all 5 suggested self-management functionalities (ie, educational information on pain and stress, a total of 87 pain-specific and stress-specific self-help skills, goal setting through tasks of the day and tasks of the week, multidimensional self-monitoring, and social support) and combines this content with gamification to motivate and engage the user. This poses great potential for supporting in-person therapy and reducing therapy costs [56].

### Motivation

In contrast to the general recommendation, most pain management apps are not based on scientific evidence and have not been thoroughly tested [39]. This also means that designers have neither included experts from pain management in the development of their apps [38] nor used expert reviews to assess their quality [34]. Contrary to this, Pain-Mentor’s contents were extended in consultation with a physician who specialized in chronic pain treatment. The involvement of health care professionals in the development of health apps, as was done for Pain-Mentor, is important for health apps to contribute value to the delivery of health care and chronic disease management [57]. With many apps promising effective pain treatment, patients face a large array of possible apps to choose from, with little guidance regarding their quality [31]. To ensure quality, functionality, and relevance of the content, health apps need to be tested in scientific trials and must involve health care professionals not only during their development but also in the evaluation process [58].

In general, health apps must be acceptable to both the user who must decide whether the program is usable and can provide benefit in an operational environment and the health professionals who determine whether the app does what it is supposed to do [59]. In contrast to the users’ goal, the experts’ primary goal is to assess the quality of a health app to identify apps that can be recommended to their patients [60]. They focus on different aspects and provide different feedback than users and developers [61]. Therefore, even though experts have scientifically evaluated few pain management apps [35], testing the quality of health apps through expert evaluations is essential to assess the quality of key app features [62].

This study, therefore, conducted an expert evaluation of the first prototype of the newly developed pain management app, Pain-Mentor, that combines a multimodal approach to pain self-management with an extensive gamification framework. The aim of this study was to gather information on how to further improve Pain-Mentor to create an app that has high value, the potential to positively influence chronic pain patients, and is accepted and recommended by medical professionals. For this purpose, the app’s general quality was evaluated from the perspective of health professionals. This approach enabled us to identify areas that need improvement and helped to further adjust Pain-Mentor for the purpose of its application.

## Methods

### Recruitment

**Figure 2:**
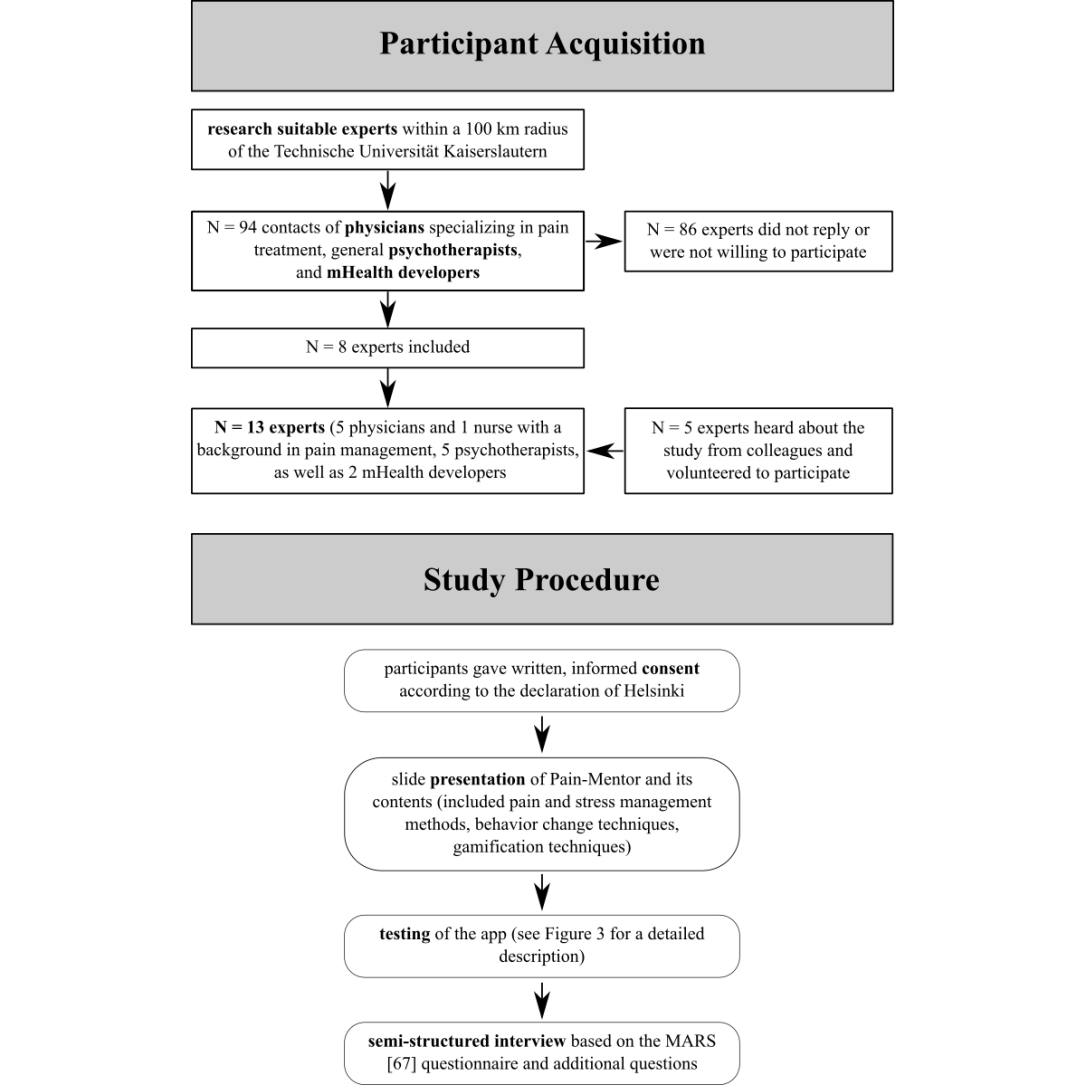
Depiction of the participant acquisition and study procedure. MARS: mobile application rating scale; mHealth: mobile health.

To assess the app’s quality per the standards of health professionals, experts with a background in chronic pain management and mHealth development were recruited (Figure 2). For this purpose, based on an internet search, physicians specializing in pain treatment and general psychotherapists in a 100-km radius of the Technische Universität Kaiserslautern (Germany) were contacted via email. Among the 94 experts contacted, 8 were willing to participate in this study. An additional 5 experts learned about the study from one of their colleagues and volunteered to participate as a result. In the end, 13 experts (5 physicians, 1 nurse with a background in pain management, 5 psychotherapists, and 2 mHealth developers) participated in this study. Previous research suggested the use of at least 2 to 4 experts [63-65], although a larger sample size increases the percentage of identified problems in the apps. As about 95% of all problems can be identified with as many as 9 participants [66], it can be concluded that the sample size of this study provided good insight into the app’s quality and enabled to identify most of the concerns arising from experts from chronic pain treatment. All participants had specific experience in the field of pain and pain management.

### Procedure

The whole procedure was approved by the local ethics committee from the Department of Social Sciences, Technische Universität Kaiserslautern.

To ensure a standardized approach, the following study procedure was predefined. At the beginning, the health professional was informed about the procedure, aim, and data collected in the study. Each participant gave written consent according to the Declaration of Helsinki. Afterward, Pain-Mentor and its contents were presented to the professional in detail in a slide presentation that explained the pain and stress management methods, behavior change techniques, and gamification aspects that were included and how they were interconnected. After the presentation, the expert tested the app (Figure 2) on a tablet (Lenovo TB-4706F). For this purpose, the app was set to a specific prerequisite to ensure all participants were exposed to the same content and features.

**Figure 3:**
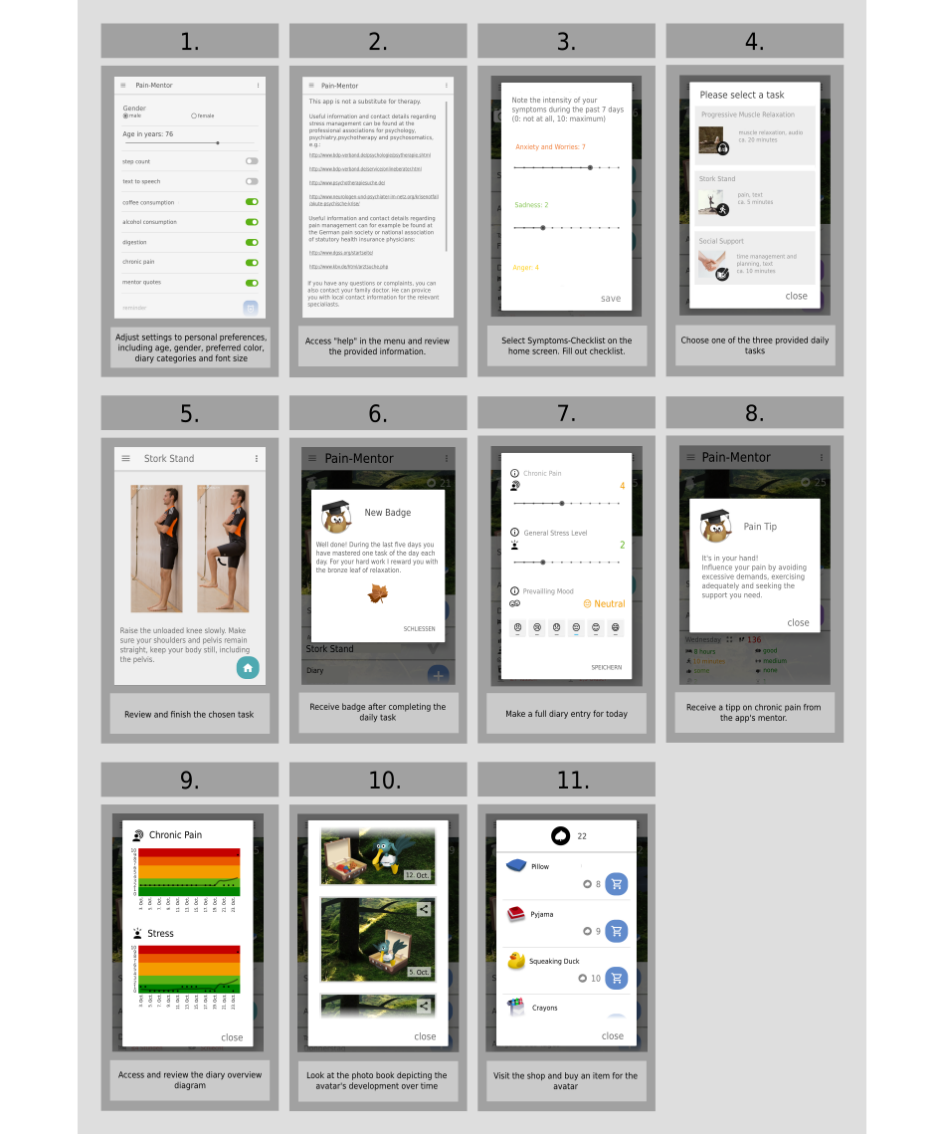
Sequential order of the app testing process that all experts followed.

Testing also followed a predefined process that took 15 to 25 min for each participant (see Figure 3 for a detailed description).

### App Quality

After testing Pain-Mentor, all experts rated the quality of the app and were asked for feedback. For this purpose, the mobile application rating scale (MARS) [67] was applied. MARS was specifically designed to assess the quality of health apps with the help of experts from health and information technology [67]. MARS comprises 6 subscales. Four of those scales (ie, engagement, functionality, esthetics, and information quality) assess the general app quality; the subjective quality section evaluates the user’s overall satisfaction; whereas the app-specific section assesses the perceived impact of the app on the user’s knowledge, attitudes, intentions to change, and the likelihood of actual change in the target health behavior. The complete structure of MARS [67] is listed in Table 1.

**Table 1:** Structure of the mobile application rating scale questionnaire Stoyanov et al (2015) [67].

Section and subsection		Definition		Items	
**App quality**					
	A: Engagement		Fun, interesting, customizable, interactive (eg, sends alerts, messages, reminders, and feedback and enables sharing), and well targeted to audience		Entertainment Interest Customization Interactivity Target group
	B: Functionality		App functioning, easy to learn, navigation, flow logic, and gestural design of app		Performance Ease of use Navigation Gestural design
	C: Aesthetics		Graphic design, overall visual appeal, color scheme, and stylistic consistency		Layout Graphics Visual appeal
	D: Information		Contains high quality information (eg, text, feedback, measures, and references) from a credible source		Accuracy of app description Goals Quality of information Quantity of information Visual information Credibility Evidence base
App’s subjective quality		Contains subjective, personal opinion of the app, including recommendation to others, estimated usage, willingness to pay, and overall star rating		Likelihood of recommending the app to others Estimated usage over the next year Willingness to pay for the app Overall star rating	
App-specific		Perceived impact of the app on the user’s knowledge, attitudes, and intentions to change as well as the likelihood of actual change in the target health behavior		Awareness Knowledge Attitudes Intention to change Help seeking Behavior change	

Participants rated each of the 23 MARS items on a 5-point Likert scale (from 1=inadequate to 5=excellent). To allow for differentiated user feedback, MARS was applied as a semistructured interview. This means, after each rating, participants had the opportunity to explain their answer and give suggestions regarding further improvement of the app (open-response format). Presenting MARS and other questionnaires as semistructured interviews has been done in previous studies and promises deeper insights into the raters’ reasoning and possible improvements (eg, the study by Anderson et al [68]). As the experts spent limited time trying out the app (approximately 20-30 min), an additional answer option was added to each question, namely, “I cannot assess this.” This ensured that the experts were not forced to answer, if they felt that they did not have enough time with the app to assess an aspect.

Overall, 3 questions from the subscale, *subjective quality*, were removed from the questionnaire: (1) question 18, “Does the app come from a credible source?” because the source of the app was explained to the participants in detail; (2) question 19, “Has the app been tested?” because an evaluation regarding the app’s effectiveness has not been conducted so far; and (3) question 22, “Would you pay for this app?” because health experts are not the target user audience of this app. In addition, question 20 was adapted to the context and changed into “Would you recommend this app to patients who might benefit from it?” The questions from the *app-specific* section were adapted to the context, ie, the term *health behavior* was replaced with *stress and pain management*.

### Additional App-Specific Questions

In addition to MARS, the participants answered 7 additional questions on a 5-point Likert scale (from 1=inadequate to 5=excellent) and 1 open-response question with regard to the expected app’s appeal for patients and specific app features. As with MARS, participants received the opportunity to explain their answers. See Multimedia Appendix 1 for a list of all additional questions.

## Results

### Mobile Application Rating Scale–Based Outcomes

Overall, experts rated Pain-Mentor to be of excellent quality (mean 4.54, SD 0.55). Subjective app quality was appraised as excellent (mean 4.57, SD 0.43). The app-specific questions were rated as good, with a mean of 4.38 (SD 0.75).

The MARS quality subsection, engagement, was rated as good, and functionality, esthetics, and information were rated as excellent (see Multimedia Appendix 2 for mean values and standard deviations).

**Figure 4:**
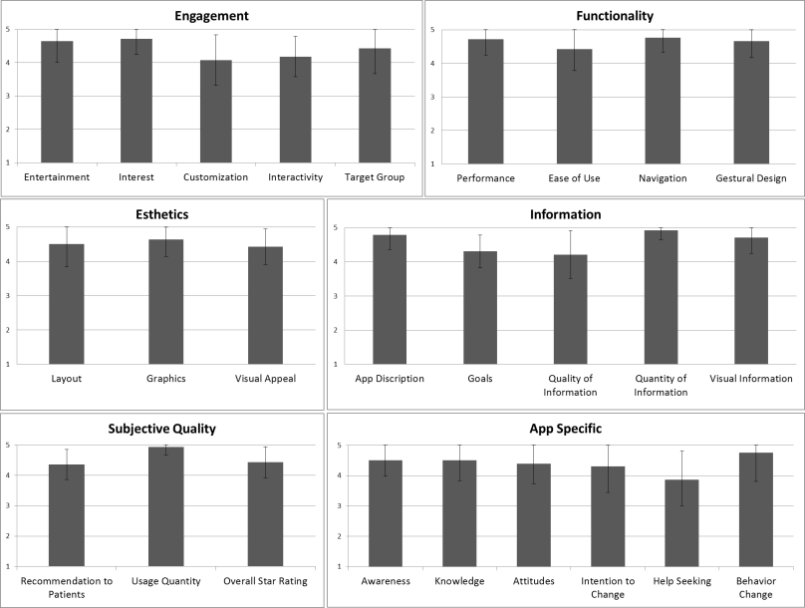
Experts’ rating (in means and standard deviations) of the mobile application rating scale regarding Pain-Mentor.

Evaluating each question of these subsections in detail, the results showed a mean value ranging from of 4.07 (SD 0.76) for customization to 4.71 (SD 0.47) for interest for the subsection engagement. Functionality showed mean rating between 4.43 (SD 0.65) for ease of use and 4.77 (SD 0.44) for navigation. The app’s esthetics were assessed as excellent, with mean values of 4.43 (SD 0.51) for visual appeal, 4.50 (SD 0.65) for layout, and 4.64 (SD 0.50) for graphics. The information communicated in Pain-Mentor also showed good to excellent ratings, with means ranging from 4.21 (SD 0.67) for the quality of information and 4.92 (SD 0.28) for information quantity. The subjective quality of the app showed ratings of 4.36 (SD 0.50) with regard to the recommendation of the app to patients and the overall star rating and 4.50 (SD 0.52) for usage duration. The app-specific questions of MARS showed values between 3.86 (SD 0.95) with regard to encouraging patients to seek help outside the app and 4.75 (SD 0.46) regarding the app’s potential to promote behavior change. A detailed visualization of the results is included in Figure 4.

Occasionally, experts were unable to answer a question (see Multimedia Appendix 3 for details). Participants gave several reasons for being unable to assess these questions, which will be discussed in the Limitations.

In addition to the quantitative ratings, applying MARS as a semistructured interview provided differentiated expert feedback. The experts’ comments are discussed in the Principal Findings section of the Discussion.

### Outcomes From Additional Questions

Good to excellent mean ratings could be observed for all additional questions (see Table 2). Experts thought that it was highly likely that Pain-Mentor would appeal to patients (mean 4.50, SD 0.52). The app’s gamification concept was rated as good (mean 4.29, SD 0.61). The experts’ expectations of Pain-Mentor were mostly fulfilled (mean 4.35, SD 0.63). They rated the implemented diary (mean 4.71, SD 0.61), daily tasks (mean 4.64. SD 0.63), and symptoms checklist (mean 4.79, SD 0.43) as sensible and useful. Moreover, they believed that using Pain-Mentor to support in-person therapy would be useful (mean 4.57, SD 0.51). All experts were able to assess all additional questions.

**Table 2:** Experts’ ratings for additional questions regarding Pain-Mentor.

Question	Rating, mean (SD)
Do you think the app would appeal to patients?	4.50 (0.52)
Did you like the gamification concept?	4.29 (0.61)
Did the app meet your expectations?	4.35 (0.63)
How useful is the diary?	4.71 (0.61)
How useful is the concept of daily exercises?	4.64 (0.63)
How useful is the symptoms checklist?	4.79 (0.43)
How useful is it to apply the app in addition to therapy?	4.57 (0.51)

## Discussion

### Principal Findings

Overall, experts rated the app to be of excellent overall and subjective quality. The app-specific section of MARS was rated as good. These positive results reflect that the app and its contents (ie, diary, daily tasks, information, symptoms checklist, and gamification concept) met the experts’ expectations. As expected, these results show that combining the 5 suggested self-management functionalities (ie, educational information, self-care skills, self-monitoring, goal setting, and social support) [7-11] in an app targeting chronic pain management is approved by health experts.

In addition, the experts thought that the app’s gamification concept, especially the avatar, made the program engaging and interesting to use. This was mirrored by the experts’ comments, eg, E4 said, “I think the app motivates those patients who want to be proactive and do something to manage their pain.” This implicates that the use of gamification could pose a solution to the lack of engagement and entertainment in pain management apps as mentioned in the study by Jamison et al [28]. It also agrees with the results from a user study with the stress management app, Stress-Mentor, which showed that the app’s gamified concept led to increased usage compared with a nongamified control group (Hoffman et al, unpublished data). Though this is a promising indication of the effectiveness of the concept of Pain-Mentor, a user study with patients with chronic pain is needed to show whether these results remain.

Two experts expressed concerns that the existing gamification concept, especially the choice in avatar, might be too childlike and not suitable for the elderly. Although most users prefer human avatars that match their own gender [69], it is very subjective as to which avatar appeals to a user. To solve this problem, the participants suggested providing the user a choice among different avatars and including more options to adapt the avatar to the user’s preferences. Despite some experts’ skepticism regarding the avatar’s suitability for the elderly, other experts thought that Pain-Mentor was well suited for the target group (ie, adults with chronic pain) and that it was very likely that the app would appeal to patients. This further supports the combination of evidence-based content and gamification [43,45-47] and indicates that such approaches should not be limited to adolescents. In addition, gamification most likely had a positive impact on the app’s esthetics.

Experts especially liked that they could choose the avatar’s color and that the avatar’s appearance was linked to both diary entries and progress (eg, E9 stated, *“*It’s nice that the user can pick the avatar’s color. Especially the visual elements invite you to explore and play around a little”), and they liked that the app’s *simple visual design* allowed them to use it intuitively*.* This backs both personalization [70] and the use of vicarious reinforcement through avatars in health apps [51].

Although the experts were mostly satisfied with Pain-Mentor’s customizability, they also suggested including more reminders to help the user remember to practice throughout the day. This shows that one reminder is the minimum, whereas the inclusion of more appears to be preferable. Nonetheless, the reminder function was generally perceived positively, and the experts thought that this feature was likely to promote the user’s self-commitment. Overall, 7 experts also suggested the addition of new diary aspects. However, there was no consensus among the experts on the aspects that should be added (they suggested, eg, weight, additional dietary aspects, pain location, and notation of additional exercises). Thus, it cannot be concluded which categories would make the most sense to be added based on the feedback obtained from this study. Moreover, the addition of aspects could easily overwhelm the users and lead to a decline in the app’s usability and usage [71]. Furthermore, 2 experts also commented that not all aspects are equally important for every patient. To solve this issue, the developers could add a notes section that leaves room for further patient-specific entries, as has been implemented in other pain management apps (eg, Chronic Pain Tracker and FitBack) [12,34]. Another approach could be to allow the addition of individual scales in the diary [72]. Both solutions would leave the choice of adding further diary categories to the user and his or her treating health professional.

Regardless of the suggested extensions, the diary was perceived as very useful by the experts. One participant expressly mentioned that she especially liked that the diary was not overly focused on pain, but rather allowed for tracking the patient’s overall well-being, including emotional (stress level and mood) and cognitive aspects (daily hassles and daily uplifts). This goes in line with previous studies [34,35] that criticized that most pain management apps focused only on tracking medication and pain levels. Another aspect that was mentioned was the advantage for patients in keeping a digital diary instead of applying a pen and paper approach [73].

Though the app’s interactivity was assessed as good, one expert commented that it could be further improved by giving advice based on the user’s diary entries. Consequently, developers should think about further personalizing their apps by linking the users’ entries (eg, from a diary) to suitable health information and tasks. For example, the app, MyBehavior, automatically provides personalized suggestions based on a health diary [74].

Participants thought that the information imparted in the app was generally well formulated and of high quality. Moreover, experts would recommend the app to many of their patients based on whether he or she would profit from using the app. This shows that the experts thought the app to be a good therapy supplement. However, their recommendation largely depends on the patients’ age, disease pattern, and current state. Nevertheless, age does not necessarily impact compliance or satisfaction with a pain management app [28]. Moreover, the combination of visual features (because of the gamification concept) and content [50] was perceived very positively (eg, E9 said, “It [the app] has a good balance of simple visual design and good content-related information”). This further supports the use of gamification in the context of pain management.

There was general approval of the self-management skills that are imparted in the app. Nevertheless, 3 experts suggested including additional tasks, such as more stretching exercises, more tasks specifically aimed at dealing with pain, and exercises aimed at distracting patients from their pain. This emphasized that experts see the potential of using apps to teach a large number of stress-related self-help skills to the user, including relaxation, problem solving, and cognitive aspects [13]. However, it should be supplemented by more pain-specific aspects to provide maximal suitability.

The experts also mentioned that they would like to be able to review the app’s data with their patients on a computer to monitor and discuss their patients’ progress. However, automatic data transmission to the treating health professionals was seen as problematic because of the experts’ limited availability of time. To avoid this problem, an optional sharing function could be added that allows patients to share their data with the health professionals on a voluntary basis [34]. Such functions provide health professionals the opportunity to gather data on a patient’s behavior [75]. My Pain Diary, eg, offers the export of data to a computer [28].

The experts’ positive ratings of the app’s ability to positively influence patients’ awareness, knowledge, attitude, intention to change, help-seeking, and behavior change are in line with the fact that health apps can enhance users’ self-management of chronic conditions [27]. However, it was emphasized that the individual played an important role regarding these aspects. Nonetheless, the experts thought that it would be very useful to employ the app to supplement in-person therapy. This highlights the potential of health apps to support regular treatment [26].

In addition to using the app for therapy support, one expert suggested to use the app to bridge the time until patients can receive in-person therapy. As waiting times for therapy are often long, using health apps in this manner could further increase their potential to improve health care [22] and diminish the number of patients who do not receive adequate care [21]. This further supports the conclusion that pain management apps could be especially beneficial in out-clinic settings [29]. Therefore, this area of application should be the focus of future research.

When asked which aspects they thought would keep patients from using the app, experts mostly mentioned a lack of motivation for people to change. However, they also mentioned that it is often difficult for patients to deal with the subject matter. In addition, they mentioned a lack of familiarity with mobile devices, apps and data security. This is in line with previous studies that have shown that technical affinity [76] and data privacy and security [77] are important aspects for choosing and using health technologies. Therefore, developers should make sure they pay special attention to data security when developing health apps [75]. Pain-Mentor, for instance, only saves the user’s data locally on his or her smartphone in an encrypted form.

Overall, Pain-Mentor’s MARS ratings are similar to those of the best-rated pain management apps reviewed by Salazar et al [39]. Averaged across all reviewed apps, the mean functionality was assessed as good, whereas Pain-Mentor received an excellent score. Similar observations can be made for esthetics (average for Salazar et al [39] and excellent for Pain-Mentor), engagement (average for Salazar et al [39] and good for Pain-Mentor), information (average for Salazar et al [39] and excellent for Pain-Mentor), subjective quality (average for Salazar et al [39] and excellent for Pain-Mentor), and app-specific scores (poor for Salazar et al [39] and excellent for Pain-Mentor).

Although the experts’ comments showed that some adjustments, such as adding more pain-specific exercises, diary categories, and reminders, could further improve the app, they still rated Pain-Mentor to be of excellent overall quality (mean rating of average for Salazar et al [39]). This shows that minor adjustments of suitable health apps (eg, from stress management) can make them useful tools that can be applied in different contexts (eg, chronic pain management). Nonetheless, whether an app will be assessed as useful or not depends on both the app’s content and the suggested context of use [78]. Not every health app should be applied in or adjusted for other contexts. Underlining the importance of expert evaluations [58], this study showed that involving health experts from the target context helps to determine an app’s suitability and to identify necessary adjustments.

### Limitations

The experts approved of the app’s gamification concept and thought that using gamification in this manner could improve patients’ motivation and engagement. As experts and patients focus on different aspects [59], a next step should be to get the opinion of patients with chronic pain to further adjust the app to their needs. Though user studies showed that the app’s gamified concept is accepted by users in the context of stress management (Hoffman et al, unpublished data and [79]), it remains unclear as to what extent the results of this study hold true in the context of pain management. In addition, further research is needed to determine the effects of gamification on users’ behavior. Moreover, although experts approved of the idea to use the app to support therapy, randomized controlled trials are needed to actually determine the effectiveness of Pain-Mentor as a therapy support tool.

The inclusion of 9 experts has been suggested to be sufficient to reveal most problems within an app [64]. Thus, although only 13 experts participated, in this study, we should have identified the most important improvements that are required. Nonetheless, for future studies, it should be noted that it can be hard to obtain experts to participate. Out of 94 contacts, only a few were willing to participate. A major reason for this was the lack of time to accommodate the study within their busy work schedule.

Other aspects that future studies might encompass include the participating experts’ affinity to technology and their knowledge in testing mobile apps. Though the participants’ affinity to technology was not systematically collected in this study, 3 participants mentioned that they were not proficient in using smartphones and tablets. This means, not all participating experts had high technological knowledge.

As all experts were given a detailed introduction of Pain-Mentor and spent approximately 20 to 30 min using the app, they received detailed insights into how the app worked. Nonetheless, experts had trouble assessing the app’s gestural design and the app’s potential to positively affect behavior change. Future studies could avoid this problem through longer trial periods.

### Conclusions

This study provided a first affirmation of Pain-Mentor’s concept. The participating health experts approved of the app’s gamification aspects and described this approach as a good way to enhance user motivation and engagement. Moreover, the app received positive ratings with regard to general and subjective quality as well as app-specific aspects (MARS). This showed that the use of gamification did not have a negative impact on the app’s credibility and integrity and that the combination of gamification with the 5 recommended self-management functionalities (ie, pain-related education, self-monitoring, goal setting, social support, and the training of self-care strategies) led to an overall positive evaluation of Pain-Mentor. This indicated that the app’s development is on the right track.

The study also showed that applying MARS in combination with additional, more app-specific questions in a semistructured interview can provide insights into the participants’ ratings and disclose possible areas for improvement. In fact, this approach revealed areas where adjustments need to be made to further tailor Pain-Mentor for its application as a support tool for chronic pain therapy. The applied approach therefore helps to adjust health apps for a specific target audience and to identify further scenarios for application.

## Appendix

Multimedia Appendix 1 List of additional questions for the semistructured expert interview.

Multimedia Appendix 2 Means and standard deviations of the experts’ ratings for each category of the mobile application rating scale questionnaire.

Multimedia Appendix 3 List of questions that at least one expert felt unable to assess and the number of experts that were unable to assess each of these questions.

## Abbreviations

MARS: mobile application rating scale
mHealth: mobile health
