# Supplementary material for: Toward Gamified Pain Management Apps: Mobile Application Rating Scale–Based Quality Assessment of Pain-Mentor’s First Prototype Through an Expert Study
Source: JMIR Form Res. 2020 May 26;4(5):e13170. doi: 10.2196/13170 (PMC7284405; doi:10.2196/13170)
Supplement: Multimedia Appendix 1 [file formative_v4i5e13170_app1.docx]

|  | **Question** | **Answer options** |
| --- | --- | --- |
| 1. | Do you think the app would appeal to patients? | N/A I cannot assess this  1 definitely not  2 rather not  3 probably  4 very likely  5 of course |
| 2. | Did you like the gamification concept? | N/A I cannot assess this  1 not at all  2 a little  3 so/so  4 mostly  5 completely |
| 3. | Did the app meet your expectations? | N/A I cannot assess this  1 not at all  2 a little  3 so/so  4 mostly  5 completely |
| 4. | How useful is the diary? | N/A I cannot assess this  1 not at all  2 not useful  3 so/so  4 mostly  5 very |
| 5. | How useful is the symptoms-checklist? | N/A I cannot assess this  1 not at all  2 not useful  3 so/so  4 mostly  5 very |
| 6. | How useful is the concept of the daily exercises? | N/A I cannot assess this  1 not at all  2 not useful  3 so/so  4 mostly  5 very |
| 7. | How useful is it to apply the app in addition to therapy? | N/A I cannot assess this  1 not at all  2 not useful  3 so/so  4 mostly  5 very |
| 8. | What do you think could keep patients from using the app? |  |
