# Supplementary material for: Toward Gamified Pain Management Apps: Mobile Application Rating Scale–Based Quality Assessment of Pain-Mentor’s First Prototype Through an Expert Study
Source: JMIR Form Res. 2020 May 26;4(5):e13170. doi: 10.2196/13170 (PMC7284405; doi:10.2196/13170)
Supplement: Multimedia Appendix 2 [file formative_v4i5e13170_app2.docx]

| **MARS Category** | **Mean** | **Standard Deviation** |
| --- | --- | --- |
| General app quality | 4.51 | 0.54 |
| Subjective Quality | 4.51 | 0.31 |
| App specific | 4.27 | 0.76 |
| Engagement | 4.32 | 0.62 |
| Functionality | 4.73 | 0.50 |
| Esthetics | 4.45 | 0.56 |
| Information | 4.53 | 0.49 |
