# Supplementary material for: Toward Gamified Pain Management Apps: Mobile Application Rating Scale–Based Quality Assessment of Pain-Mentor’s First Prototype Through an Expert Study
Source: JMIR Form Res. 2020 May 26;4(5):e13170. doi: 10.2196/13170 (PMC7284405; doi:10.2196/13170)
Supplement: Multimedia Appendix 3 [file formative_v4i5e13170_app3.docx]

| **Question** | **N** |
| --- | --- |
| Customization | 1 |
| Navigation | 1 |
| Goals | 1 |
| Information quantity | 1 |
| Attitudes | 1 |
| Intention to change | 1 |
| Awareness | 2 |
| Interactivity | 3 |
| Gestural design | 5 |
| Behavior change | 6 |
